# A comprehensive evaluation of oversampling techniques for enhancing text classification performance

**DOI:** 10.1038/s41598-025-05791-7

**Published:** 2025-07-01

**Authors:** Salimkan Fatma Taskiran, Bahaeddin Turkoglu, Ersin Kaya, Tunc Asuroglu

**Affiliations:** 1 Department of Computer Engineering, Konya Technical University, Konya, 42250 Turkey; 2 Department of Artificial Intelligence and Data Engineering, Ankara University, Ankara, 06830 Turkey; 3 Faculty of Medicine and Health Technology, Tampere University, Tampere, 33720 Finland; 4 VTT Technical Research Centre of Finland, Tampere, 33101 Finland

**Keywords:** Imbalanced datasets, Text classification, Synthetic minority over-sampling technique (SMOTE), Computer science, Computational science

## Abstract

Class imbalance is a common and critical challenge in text classification tasks, where the underrepresentation of certain classes often impairs the ability of classifiers to learn minority class patterns effectively. According to the “garbage in, garbage out” principle, even high-performing models may fail when trained on skewed distributions. To address this issue, this study investigates the impact of oversampling techniques, specifically the Synthetic Minority Over-sampling Technique (SMOTE) and thirty of its variants, on two benchmark text classification datasets: TREC and Emotions. Each dataset was vectorized using the MiniLMv2 transformer model to obtain semantically rich representations, and classification was performed using six machine learning algorithms. The balanced and imbalanced scenarios were compared in terms of F1-Score and Balanced Accuracy. This work constitutes, to the best of our knowledge, the first large-scale, systematic benchmarking of SMOTE-based oversampling methods in the context of transformer-embedded text classification. Furthermore, statistical significance of the observed performance differences was validated using the Friedman test. The results provide practical insights into the selection of oversampling techniques tailored to dataset characteristics and classifier sensitivity, supporting more robust and fair learning in imbalanced natural language processing tasks.

## Introduction

Imbalanced datasets represent a significant challenge commonly encountered in real-world problems. In the field of classification problems, imbalanced datasets arise when the numbers of samples representing different classes varies substantially^1^. Such datasets can adversely impact the training process of classifier models. As the model tends to focus on the majority class during training, it may fail to adequately learn the minority classes. This issue becomes particularly pronounced in problems where certain classes have very few instances. Consequently, although the overall accuracy of the model may appear high, its performance in correctly classifying the minority class remains insufficient.

Classification models typically operate under the assumption that each class within a dataset contains an equal number of instances. However, this assumption becomes inadequate when the minority class holds greater importance than others. Developing highly effective classifiers for such imbalanced datasets remains a significant challenge. As the imbalance in the dataset increases, the model tends to overfit the majority class, impairing its ability to achieve the desired performance on minority classes and leading to biased outcomes. Hence, the reliability of the model in real-world applications deteriorates, resulting in failures in critical scenarios where the accurate detection of minority classes is essential^2^. The classification of text data frequently encounters severe class imbalance challenges in critical real-world applications, including hate speech detection, cyberbullying identification, fraud detection in communication systems, and sentiment analysis of underrepresented viewpoints^3–9^. In these domains, failure to accurately detect minority classes can result in serious ethical, legal, and operational consequences. By systematically evaluating a broad range of SMOTE-based oversampling methods, this study offers a practical framework for enhancing classifier robustness and promoting fairness in imbalanced natural language processing tasks.

Imbalanced datasets constitute a prevalent challenge across various domains, irrespective of data type. In text classification, this imbalance often stems from the natural dominance of certain topics or sentiments over less frequent ones^10^. For instance, in major natural language processing tasks such as sentiment analysis, positive reviews may substantially outnumber negative ones, resulting in an imbalanced dataset^11^. This situation may cause models to preferentially learn majority classes while neglecting the minority ones. Several strategies have been developed to achieve successful outcomes when working with imbalanced datasets. Among these, oversampling — wherein synthetic data is generated for the minority class to balance the dataset — remains one of the most widely adopted approaches^12^. Conversely, undersampling — which reduces the number of samples from the majority class to achieve balance — is generally less favored as it results in data loss and is more typically employed in specific problems settings^13^. In addition to approaches that modify the dataset, successful models can also be developed using algorithms that exhibit reduced sensitivity to class imbalance^14^. These algorithms may consist of modified versions of existing methods that have been enhanced for greater robustness against imbalance, or entirely novel algorithms specifically designed for this purpose^15^.

Text classification is a machine learning task frequently encountered within the field of natural language processing. It can be defined as the assignment of a text document to a specific category or class. Common examples include classifying an email as spam or not spam, identifying the topic of an article, or detecting the sentiment of a tweet. The stages of text classification necessitate the application of various key natural language processing techniques. A typical text classification pipeline comprises multiple stages: text cleaning, preprocessing (e.g., tokenization, stopword removal), normalization (e.g., stemming or lemmatization), feature extraction or vectorization, and finally classification. Each stage entails methodological choices that can substantially impact downstream performance. For instance, while stemming accelerates processing by reducing words to their base forms, lemmatization yields linguistically accurate root forms, potentially enhancing semantic consistency. Similarly, vectorization methods range from traditional frequency-based techniques (e.g., TF-IDF) to modern deep contextual embeddings (e.g., BERT, MiniLMv2), each presenting trade-offs between computational cost and representational richness^16–18^. These methodological decisions become particularly critical when addressing imbalanced datasets, where subtle differences in representation can exacerbate classification bias or variance.

In this study, the impact of imbalanced datasets on text classification, a fundamental problem in natural language processing, was investigated. After vectorizing the two datasets, class balancing was performed on the resulting representations. SMOTE (Synthetic Minority Over-sampling Technique) and its variants were employed as the oversampling methods for balancing. The vectorized and balanced datasets were subsequently classified using various machine learning algorithms, and their performance was compared and analyzed against the results obtained from the original datasets. Furthermore, the effectiveness of different oversampling methods was evaluated in terms of their impact on classifier performance.

To the best of our knowledge, this study constitutes the first systematic benchmarking of thirty one widely used SMOTE-based oversampling techniques on two benchmark text classification datasets (TREC and Emotions), utilizing transformer-based vectorization. By implementing a consistent pipeline across six classifiers and multiple resampling strategies, this study fills a crucial gap in the literature and provides new insights into selection of effective data augmentation methods for handling textual imbalance problems.

The remainder of the article is organized as follows: Sect. 2, titled *Related Work*, reviews the existing literature on imbalanced data and text classification. Section 3, titled *Experimental Setup*, provides a detailed description of the experimental design and procedures. Section 4, titled *Experimental Results*, presents the study’s findings along with their interpretation. Finally, Sect. 5, titled *Conclusion*, discusses insights gained from the study, offers recommendations for extending the research, and suggests directions for further studies.

## Related work

### Imbalanced data

The problem of imbalanced datasets constitutes a prevalent challenge, particularly in studies focused on anomaly detection^19^. In real-world applications, numerous problems involve anomaly detection including disease diagnosis in the medical field, malfunction detection in industrial machinery, fraud detection in the financial sector and text classification^20–23^. Learning from imbalanced datasets remains as one of the fundamental challenges in many real-world machine learning applications. Various strategies have been proposed to address the class imbalance problem, with resampling techniques being among the most widely adopted approaches^24^. These methods aim to enhance classifier performance by balancing class distribution during the imbalanced learning process, either by adjusting the minority or majority class. Resampling methods typically operate in two main ways: the first involves removing samples from the majority class, known as Random Undersampling^19^ while the second one involves adding new samples to the minority class, known as Random Oversampling. However, the resampling process carries the risk of losing critical information relevant to the dataset and the underlying problem. To mitigate such risks, improved versions of these algorithms have been proposed.

The Synthetic Minority Over-sampling Technique (SMOTE) is an advanced oversampling method that generates new data points by creating synthetic examples between neighboring instances of each sample in the minority class^25^. As one of the most widely used oversampling methods today, SMOTE also forms the foundation for the development of numerous subsequent oversampling techniques^26^. SMOTE generates synthetic data by interpolating between pairs of minority class instances. This approach helps preserve the original characteristics of the minority class, introduces variability, and expands the decision boundary by enriching the feature space with additional representative samples.

Despite its widespread adoption, SMOTE exhibits several limitations. To address these shortcomings, various extensions have been proposed. Borderline-SMOTE^27^ focuses on minority instances near the decision boundary, selectively generating synthetic samples in these regions to improve classification performance. Safe-Level SMOTE^28^ introduces a safety score based on local minority density, aiming to reduce the risk of generating noisy samples near majority class regions. ADASYN^29^ further refines this strategy by adaptively generating a greater number of synthetic samples for minority instances that are harder to learn.

More advanced SMOTE variants incorporate clustering, ensemble methods, and data cleaning strategies. Cluster-based approaches, such as Cluster-SMOTE^30^ and DBSMOTE^31^, apply SMOTE within clusters formed by algorithms like K-means or DBSCAN to better preserve the internal structure of the minority class. Ensemble-based methods including SMOTEBoost^32^, RUSBoost^33^, and EUSBoost^34^ integrate oversampling or undersampling into boosting frameworks to improve classifier focus on hard-to-learn instances. Additionally, hybrid techniques such as SMOTE-ENN and SMOTE-Tomek^35^ combine oversampling with data cleaning techniques to refine class boundaries.

### Text classification

Today, text-based data constitutes a substantial portion of big data repositories. Effectively structuring and classifying text-based data have become critically important tasks. Successfully accomplishing these tasks not only saves time and resources but also facilitates improved information discovery and enhances decision-making processes^36^.

The study conducted by Mirończuk et al. (2018) defined six fundamental components of text classification and elaborated on various concepts underlying modern text classification. The authors systematically performed a qualitative analysis to identify both traditional and contemporary techniques across all stages of text classification process. Furthermore, they examined prevailing research trends in this field^37^.

A comprehensive study conducted by Kowsari et al. (2019) provides a general overview of the text classification problem and discusses existing algorithms, techniques, text feature extraction methods, and dimensionality reduction techniques. The authors also examined the limitations of each technique in real-world applications^38^.

For effective text classification, the features extracted from the text must possess high representational power. A study conducted by Deng et al. (2018) emphasized the importance feature selection process in text classification tasks. The authors investigated the impact of bag-of-words model and local - global dictionaries on feature selection, and further analyzed the influence of similarity metrics such as Euclidean distance, Jaccard coefficient, Pearson correlation, and cosine similarity on classification performance^39^.

In 2020, Shah et al. conducted a comparative analysis of logistic regression, random forest, and K-nearest neighbors (KNN) models for text classification. In this study, they performed an in-depth evaluation of commonly used algorithms, providing a comprehensive assessment of the effectiveness of various text classification approaches^40^.

With the widespread adoption of deep learning and the emergence of large language models, text classification problems have evolved across multiple dimensions. In 2020, Minaee et al. compared deep learning models with traditional machine learning approaches for text classification tasks such as sentiment analysis and news categorization. The authors examined 150 different deep learning models to analyze their impact on these applications^41^.

Various methods are employed to balance the minority class in text-based datasets exhibiting class imbalance. In 2022, Pellicer et al. conducted a comprehensive review of data augmentation studies in the field of natural language processing^42^. The authors categorized data augmentation techniques applied to textual data into two broad groups: data-based and feature-based methods. Data-based methods involve the direct generation of synthetic data from raw text. These modifications can occur at multiple levels, ranging from character to document level, and may include transformations at the word or phrase level. Feature-based methods, by contrast, encompass performed during or after the feature extraction process.

In 2021, Shaikh et al. generated synthetic data for highly imbalanced text datasets using GPT-2 and LSTM-based text generation models to achieve class balancing^43^. Experiments conducted on three distinct, highly imbalanced datasets from various domains demonstrated that classification performance significantly improved when data balancing techniques were applied, compared to using the same deep neural network models without balancing. This study highlights the critical importance of data balancing techniques in classification tasks involving imbalanced datasets.

In 2022, Henning et al. conducted a study on class imbalance issues in deep learning-based natural language processing tasks^44^. They specifically focused on approaches proposed for handling imbalanced data in natural language processing problems. The methods analyzed were categorized into sampling, data augmentation, loss function selection, incremental learning, and model design.

Recent studies conducted in 2023 and 2024 have brought significant advancements to the field of text classification. In 2023, Li et al. examined the potential and limitations of generating synthetic data using large language models (LLMs). Their research analyzed the effectiveness of synthetic data in classification tasks and identified key factors influencing classification performance^45^.

In 2024, Wang et al. developed novel methods based on Transformer architectures to enhance the effectiveness of text classification models. This study particularly focused on improving the understanding and classification of complex text structures^46^. Another study conducted in 2024 by Amasyalı et al. investigated the effects of data augmentation and curriculum strategies on NLP models. In this study the authors proposed a new curriculum strategy that significantly improved model performance^47^.

The issue of class imbalance in text classification has garnered significant academic attention due to its detrimental effects on model performance, particularly in domains like hate speech detection, spam filtering, sentiment analysis, and cyberbullying identification. Numerous studies have focused on improving model performance by addressing this challenge. For instance, one study utilized feature engineering to detect cyberbullying on Twitter, demonstrating that enhanced feature representations can help models better manage imbalanced data^48^. Another study applied ordinal classification methods for hate speech detection, achieving improved results on imbalanced datasets^49^. Kumar and Bhat^50^ investigated the impact of class imbalance on machine learning models for cyberbullying detection, emphasizing its negative influence on model outcomes. Additionally, Kumar et al.^51^ conducted a study on spam and cyberbullying detection using deep learning techniques, highlighting class balancing as a critical step for enhancing model performance.

Oversampling techniques such as SMOTE have proven effective across various natural language processing tasks. Singgalen and Wahyuningtyas^52^ applied SMOTE to enhance Support Vector Machine (SVM) based classification of travel vlogs, demonstrating its effectiveness on real-world data. Tchokote and Tagne^53^ showcased the power of combining SMOTE with principal component analysis (PCA) and adversarial learning to detect hate speech from multimodal sources. Afuan et al.^54^ employed SMOTE with a Naive Bayes classifier for sentiment analysis of political events, reporting significant gains in performance. In 2025 Primandari and Ermayani addressed gender-based violence classification by integrating SMOTE with XGBoost, achieving notable improvements in classification outcomes^55^.

## Material and method

In this section, the methods employed to address the issue of imbalanced data — commonly encountered in the field of text classification which constitutes the focus of this study — are discussed alongside their implementation processes. For this purpose, two English-language datasets, namely the TREC (Text REtrieval Conference) Question Classification dataset and the Emotions datasets were utilized. To mitigate the problem of class imbalance, oversampling techniques were chosen. This approach aims to achieve class balance by increasing the number of samples in the minority classes to match those of the majority class.

**Fig. 1:**
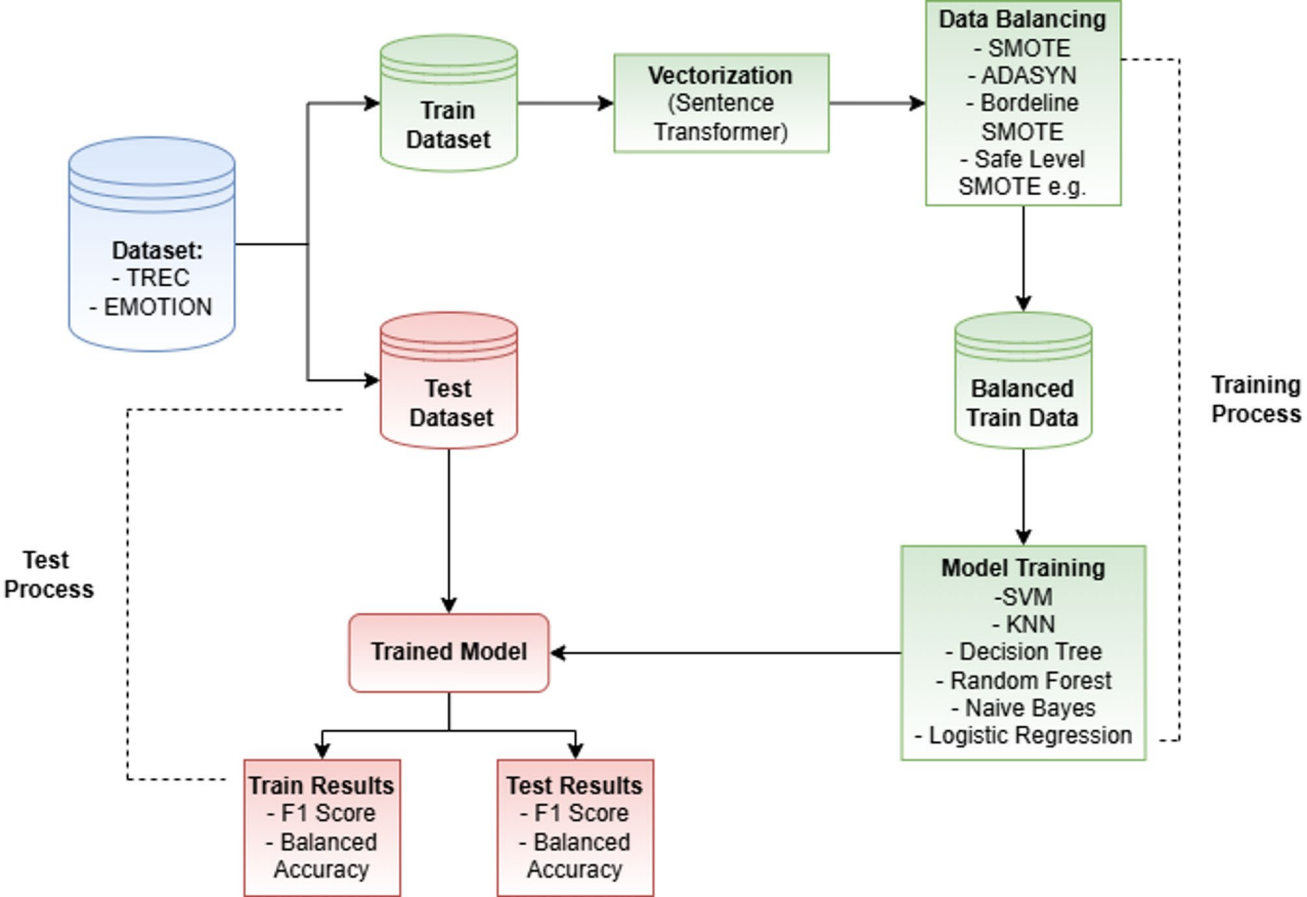
Overview of the text classification pipeline including vectorization, data balancing and model training with several classifiers.

This part of the study outlines the steps undertaken to understand and address the issue of imbalanced data. Furthermore, it examines the impact of the applied methods on classification performance. The flowchart illustrating the experimental process is presented in Fig. 1. The experimental setup is described step by step in the subsequent sections.

In the initial stage, textual data are collected from the TREC and Emotions datasets. These datasets contain pre-labeled instances to be used for the training and testing of text classification models. The preparation and preprocessing phases involve cleaning, normalization, and vectorization of the texts. This process constitutes a critical step toward enhancing performance of classification algorithms.

In the second stage, data augmentation is applied to the vectors obtained from the text data. This procedure, conducted exclusively on the training data, aims to balance the number of samples across classes by increasing the sample size of minority classes to match that of the majority class. Various algorithms are employed for data augmentation, and the classification performance associated with each algorithm is evaluated separately. Data augmentation is performed using widely adopted SMOTE algorithm and its variants.

In the next stage, classification models were trained separately on imbalanced and balanced training datasets. The primary objective of this stage is to observe the impact of data balancing on classification performance. Furthermore, the effects of different balancing methods were analyzed in detail using various classification algorithms. For this purpose, six different classifiers were employed: Support Vector Machines (SVM), K-Nearest Neighbors (KNN), Decision Trees, Naive Bayes, Logistic Regression, and Random Forest.

The final stage involves the testing of the trained models. In this phase the test data were subjected only to vectorization and subsequently classified by trained models. To evaluate classification performance, commonly used metrics for imbalanced datasets, such as F1-Score and Balanced Accuracy, were selected.

### Dataset

In the experimental study, two benchmark datasets, TREC^56^ and Emotions^57^, were utilized.

#### TREC dataset

This dataset consists of factual questions organized into six distinct categories based on broad topics. It contains 5452 labeled questions as training and 500 labeled questions as test data. The average sentence length is 10 words, and the vocabulary size is 8700 unique terms. The class distribution of the dataset is presented in Table 1.

**Table 1:** TREC dataset.

Label	Train (5452)	Test (500)	Class distribution (%)
ABBR - Abbreviation	86	9	1.5
DESC - Descriptions	1250	94	21.3
ENTY - Entities	1162	138	22.9
HUM - Humans	1223	65	22.4
LOC - Location	835	81	15.3
NYM - Numeric	896	113	16.3

#### Emotions dataset

The second dataset selected for the experimental study is the emotion dataset, designed for emotion recognition tasks. This dataset contains texts representing specific emotions. This data set is widely used in natural language processing applications such as emotion recognition and emotion analysis. The emotions in the texts are categorized into six distinct classes: Anger, Fear, Joy, Love, Sadness and Suprise. It comprises 16,000 labeled texts for training and 500 labeled texts for testing. The class distribution of the Emotions dataset is presented in Table 2.

**Table 2:** Emotions dataset.

Label	Train (16k)	Test (500)	Class distribution (%)
Anger	4666	581	13.75
Fear	5362	695	10.6
Joy	1304	159	35.2
Love	2159	275	8.9
Sadness	1937	224	27.5
Surprise	572	66	4.05

The TREC and Emotions datasets used in this study provide predefined training and testing splits. These original partitions were preserved throughout the experiments to ensure comparability with prior work and to maintain experimental integrity. No additional k-fold cross-validation was performed. All oversampling methods were applied exclusively to the training data to prevent data leakage, and model performance was evaluated on the fixed test set using macro-averaged F1-Score and Balanced Accuracy (BA).

These two datasets were intentionally selected due to their complementary characteristics. The TREC dataset focuses on fact-based question classification with moderate class imbalance, whereas the Emotions dataset involves nuanced, affect-driven categories exhibiting pronounced imbalance. This selection enables the evaluation of the generalizability of oversampling methods across both syntactic (factual) and semantic (emotive) textual domains. Moreover, both datasets are widely recognized benchmarks in text classification research, ensuring the relevance and reproducibility of the study within the NLP community.

### Vectorization

First, the datasets were subjected to vectorization. In this process, the second version of the MiniLM transformer model was employed. MiniLM is a pre-trained large language model capable of converting sentences into meaningful numerical representations^58^. The first version of MiniLM, developed in 2019, was introduced as a smaller and more efficient alternative to larger language models such as BERT. MiniLMv2, an improved version of MiniLM, was released in 2021^59^. This model is particularly effective in generating meaningful and compact vector representations from textual data.

Unlike BERT models, MiniLM employs a deep self-attention distillation technique. This technique enables MiniLM to achieve higher performance on various challenging NLP tasks while being significantly smaller and faster architecture. The self-attention mechanism is utilized to determine the relationships between words, with these relationships calculated through mathematical operations. MiniLM features an enhanced distillation process that compresses the capabilities of large language models into a more compact structure. Consequently, it can operate efficiently and effectively while requiring fewer computational resources.

MiniLM transfers the knowledge of a large model to a smaller model through the teacher-student distillation method. In this process, the teacher model functions as a large and powerful language model, while the student model is designed to be smaller and more lightweight. Despite its reduced size, the student model is capable of achieving performance levels comparable to those of the teacher model.

**Fig. 2:**
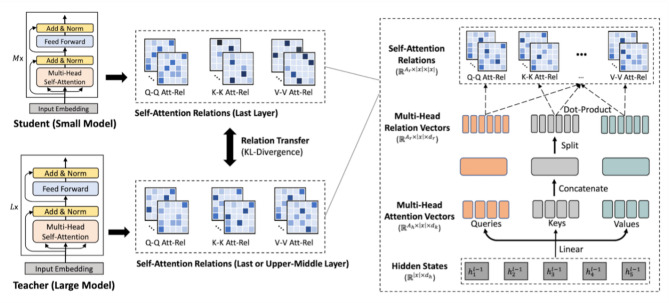
MiniLM model^58^.

This study employed the MiniLMv2 transformer model for sentence-level vectorization, which generates compact and semantically rich contextual embeddings. This approach enables more effective semantic representation compared to traditional methods such as TF-IDF and Word2Vec, and has demonstrated strong performance in text classification tasks. MiniLMv2 was selected for its balance of accuracy and computational efficiency, making it particularly suitable for large-scale experiments involving multiple oversampling techniques. The architecture of MiniLMv2 provides significant advantages in terms of computational efficiency and processing speed. The distillation process underlying MiniLM’s teacher-student model is illustrated in Fig. 2.

### Oversampling

In this study, oversampling methods were preferred over undersampling techniques due to several key factors relevant to text classification tasks. Undersampling methods reduce the number of majority class instances, potentially resulting in the loss of valuable semantic information—particularly critical in text datasets where each sample may capture unique linguistic patterns. Moreover, text classification typically involves sparse and high-dimensional feature spaces, and removing data from such spaces may compromise class separability and degrade generalization performance. In contrast, oversampling techniques preserve all original data while enriching the representation of minority classes, particularly around decision boundaries. These characteristics make oversampling more suitable and effective for addressing class imbalance in natural language processing applications. Nevertheless, it is acknowledged that undersampling may offer advantages in specific scenarios, such as when datasets contain excessive redundancy or when computational efficiency is paramount, as in streaming or real-time applications^23,60^.

In this stage of the study, oversampling strategies was adopted, where the number of data points in the minority classes is equalized to that of the majority classes. Utilizing the vectors obtained through the MiniLM model, new synthetic vector data for the minority classes were generated via oversampling. For this purpose, the widely used and well-established SMOTE method and its various extensions were employed.

The SMOTE method, proposed in 2002, selects points near minority class instances in the vector space as new samples to capture essential features of minority classes and generates synthetic data that closely resemble real instances^25^. The working principle of the SMOTE method is outlined as follows:

- A random instance $\:{x}_{i}$ is selected from the minority class,
- The k nearest neighbors of this instance are identified within the feature space,
- For each selected neighbor, a line is drawn between the original instance $\:{x}_{i}$ and its neighbor $\:{x}_{i}^{\left(k\right)}$ in the feature space,
- New synthetic data points are generated at random locations along these lines between the two original instances.

The SMOTE method employs the following mathematical formula to generate a synthetic data point between two data instances:

1 $$\:{x}_{new}={x}_{i}+\lambda\:*\left({x}_{i}^{\left(k\right)}{-x}_{i}\right)$$

Here:

- $\:{x}_{new}$, represents the newly generated synthetic data point,
- $\:{x}_{i},$ denotes the randomly selected instance from the minority class,
- $\:{x}_{i}^{\left(k\right)}$ refers to one of the nearest neighbors of $\:{x}_{i}$,
- $\:\lambda\:$ is a randomly selected value from the interval [0,1].

This process is repeated for each minority class instance until the number of data points for that class reaches the level of the majority class.

The most significant advantage of the SMOTE method is its ability to elimate class imbalance. By increasing the number of samples in the minority class, it enables the model to learn minority class patterns more effectively and make more balanced predictions. Additionally, SMOTE enhances data diversity as synthetic samples may cover new regions of feature space that are not represented in the existing dataset, thereby improving the model’s generalization capabilities. Another notable advantage is reduction of overfitting risk. By increasing the number of minority class samples, SMOTE helps mitigate the tendency of models to overfit to the majority class.

However, SMOTE also has several disadvantages. Synthetic samples may not perfectly align with real data, potentially distorting the true data distribution. This distortion can reduce the model’s adaptability to real-world scenarios. Additionally, SMOTE may result in information loss. Synthetic samples often carry less informative content compared to real data, thereby limiting the model’s learning capacity and negatively impact its performance. Finaly, SMOTE can increase data complexity; the addition of synthetic samples may raise overall of the complexity of the dataset and complicate the model’s training process.

Considering these advantages and disadvantages, the effects of using SMOTE should be carefully evaluated. It is essential to select the most appropriate data augmentation method for a given dataset and problem context.

SMOTE variants offer different strategies to address class imbalance by enhancing the basic SMOTE algorithm or hybridizing it with other techniques. Borderline-SMOTE focuses on selecting samples near the minority class boundaries to generate synthetic instances. ADASYN (Adaptive Synthetic Sampling) assigns weights to the minority class samples and generates additional samples, thus giving more attention to harder-to-learn instances. SMOTE-ENN (SMOTE with Edited Nearest Neighbors) refines the dataset by removing misclassified examples from the majority class after synthetic samples are generated. These variants aim to mitigate the limitations of the original SMOTE method and better align with the specific characteristics of the dataset to address class imbalance. In this study, alongside the basic SMOTE method, the variants listed in Table 3 were employed.

In this study, alongside the basic SMOTE method, the variants listed in Table 3 were employed. Among the 85 available oversampling techniques in the SMOTE-variants library, we selected 31 representative methods that span diverse algorithmic families to ensure comprehensive coverage. These include baseline techniques such as SMOTE and ADASYN; borderline and density-aware methods such as Borderline SMOTE and Safe Level SMOTE; hybrid and cleaning-based strategies like SMOTE-ENN and SMOTE-Tomek; clustering and manifold-oriented methods such as Cluster SMOTE and LLE SMOTE; as well as meta-ensemble and advanced sampling techniques including Assembled SMOTE and ADOMS. This selection was designed to represent a wide spectrum of methodological approaches and complexity levels within the domain of data augmentation for imbalanced learning.

**Table 3:** SMOTE variants.

Number	Method name	Description
1	SMOTE [18]	Generates synthetic data by using nearest neighbors to replicate minority class instances. It creates new data points between a selected minority class instance and its neighbors, thereby balancing the dataset.
2	ADASYN [22]	Adaptive synthetic sampling approach for imbalanced learning (ADASYN), creates synthetic data by assigning weights to minority class instances, giving more importance to less represented examples and adaptively correcting imbalances in the dataset.
3	Borderline SMOTE 1 [20]	Selects instances located near the minority class boundaries and generates synthetic samples using these instances. It is designed to improve model performance in regions where class boundaries are critical.
4	Borderline SMOTE 2 [20]	A variant of Borderline SMOTE 1 that selects borderline instances located in safer regions, aiming to enhance overall classification performance at decision boundaries.
5	SMOTE Tomek Links [28]	After generating new samples via SMOTE, the Tomek Links method is applied to remove majority class instances that are very close to the minority class, thereby refining the decision boundary.
6	SMOTE-ENN [28]	Combines SMOTE with the Edited Nearest Neighbors (ENN) technique, which removes misclassified instances from the majority class after oversampling, helping to clean the dataset.
7	Safe level SMOTE [21]	Generates synthetic samples by selecting instances from safe regions within the minority class—areas with a low risk of misclassification.
8	SMOTE RSB [54]	Achieves data balance by integrating random subset balancing with SMOTE to promote more homogeneous distribution across classes.
9	SMOTE IPF [55]	Addresses data imbalance by applying the Iterative Partitioning Filter (IPF) to eliminate the noisy data prior to oversampling, thereby improving the reliability of generated samples.
10	Lee [56]	Utilizes the nearest neighbors to generate minority class instances that better reflect the global structure of the minority class, producing more reliable and representative synthetic samples.
11	SMOBD [57]	Generates synthetic samples by minimizing deterioration, thereby ensuring the preservation of data quality and the realism of synthetic instances.
12	G-SMOTE [58]	A generalized SMOTE method that generates synthetic samples using generalized feature representations, producing instances that better reflect the overall structure of the dataset.
13	CCR [59]	Combined cleaning and resampling (CCR), combines SMOTE with a rule-based sampling approach to generate more consistent data points by applying rule-based strategies during sample generation.
14	LVQ-SMOTE [60]	Selects minority class samples using Learning Vector Quantization (LVQ) and generates synthetic instances accordingly. LVQ functions as a pattern recognition and data clustering algorithm, guiding the selection of samples for augmentation.
15	Assembled_SMOTE [61]	Integrates multiple SMOTE variants to generate synthetic samples for the minority class, leveraging the strengths of various methods to achieve more effective and robust data augmentation.
16	Polynom fit SMOTE [62]	Generates synthetic data using polynomial fitting, allowing the method capture complex relationships within the dataset and produce more representative synthetic samples.
17	LLE SMOTE [63]	Generates synthetic samples using the Locally Linear Embedding (LLE) technique, which enables the mapping of high-dimensional data onto a lower-dimensional manifold while preserving local relationships.
18	Distance SMOTE [64]	Generates synthetic samples based on distance metrics, creating new data points by leveraging the distances between minority class samples.
19	SMMO [65]	Selecting Minority examples from Misclassified data for Over-sampling (SMMO), provides a more systematic and informed balancing of the dataset.
20	ADOMS [66]	Adaptive Over-sampling Method (ADOMS) generates synthetic examples adaptively, adjusting to the dynamic characteristics of the dataset to achive more effective data augmentation.
21	MSMOTE [67]	A modified version of the SMOTE algorithm, specifically developed to handle more complex and heterogeneous datasets.
22	DE-Oversampling [68]	Generates synthetic examples using the Differential Evolution (DE) algorithm, an evolutionary computation method for solving optimization problems.
23	MSYN [69]	Margin-guided Synthetic Over-sampling (MSYN), enhances model learning by generating synthetic examples guided by margin-based rules, aligning data augmentation with the underlying data distributions.
24	SVM-balance [70]	Identifies the support vectors of the minority class using Support Vector Machines (SVM) and generates new synthetic examples around these vectors.
25	TRIM-SMOTE [71]	Searches for precise minority region while maintaining its generalization by iteratively filtering out irrelevant majority class instances from the minority region.
26	SL-Graph SMOTE [72]	Produces synthetic examples for the minority class using the Safe Level (SL) Graph method, selecting instances located at safer regions, ensuring more reliable data augmentation.
27	Random oversampling [73]	Balances the dataset by randomly duplicating minority class examples, though it carries a heightened risk of overfitting.
28	ROSE [74]	Random Over-Sampling Examples (ROSE) generates random synthetic examples for the minority class, aiming to address the dataset imbalance by introducing random samples.
29	Minority driven [75]	Focuses on the data characteristics of the minority class to produce synthetic examples that reflect the minority distribution more accurately.
30	Majority data oversampling [76]	Reduces data imbalance by decreasing the number of majority class instances, thereby balancing the dataset through selective undersampling.
31	Cluster oversampling [77]	Generates synthetic samples based on natural clusters within the dataset, leveraging clustering structures to improve the effectiveness of data augmentation.

Among recently proposed oversampling techniques, Cluster-Based Reduced Noise SMOTE (CRN-SMOTE) introduces a three-phase approach designed to enhance synthetic data quality through noise filtering and cluster preservation. This method initially applies SMOTE to increase the representation of the minority class. It then removes noisy samples using a density-based clustering algorithm, imposing an additional constraint that each class should form at most two clusters to preserve the structural coherence of class distributions. A final oversampling step is subsequently applied to the cleaned data. This framework ensures that synthetic data generation respects intra-class geometry and avoids over-fragmentation—an important distinction from SMOTE variants such as SMOTE-ENN and SMOTE-Tomek Link, which do not impose such clustering constraints. Although not empirically validated within this study, CRN-SMOTE represents a promising advancement in hybrid noise-aware oversampling strategies^61^.

### Classification & evaluation

In this part of the study, different classification models were trained on both the imbalanced and balanced versions of the training datasets. The following six traditional classifiers were employed in our experiments to evaluate the performance of the oversampling techniques: Logistic Regression (LR), Support Vector Machines (SVM), k-Nearest Neighbors (kNN), Decision Trees (DT), Naive Bayes (NB), and Random Forest (RF). These classifiers were selected to represent a diverse range of model families—including linear, probabilistic, rule-based, ensemble, and instance-based approaches—thereby ensuring a robust evaluation across different learning paradigms in the context of imbalanced text classification.

The final stage of the study involves testing the trained models. In this phase, the vectorized test data are classified by pre-trained models. To evaluate classification performance, F1-Score and Balanced Accuracy (BA) —both widely used metrics for imbalanced datasets—were employed. The F1-Score captures the balance between precision and recall, making it particularly suitable for scenarios where false positives and false negatives carry different costs. Balanced Accuracy addresses class imbalance by averaging the recall across all classes, providing a fairer evaluation under skewed class distributions. All metrics were reported using macro-averaging to ensure that each class contributed equally to the overall evaluation, regardless of its frequency^62^.

F1-Score Metric:

The F1-Score is defined as the harmonic mean of Precision and Recall values, and is commonly employed in classification problems. Its mathematical formulation is presented in Eq. (2):

2 $$\:precision = \:\frac{{True\:Positive}}{{True\:Positive + False\:Positive}},\:recall = \:\frac{{True\:Positive}}{{True\:Positive + False\:Negative}},\:F1\:Score = \frac{{2*precision*recall}}{{precision + recall}}$$

F1-Score evaluates the performance of both Precision and Recall in a balanced manner. In imbalanced classification problems, accuracy alone should not be relied upon, as it can be misleading when there are unequal numbers of instances across classes. The F1-Score achieves a high value when both Precision and Recall are simultaneously high. Therefore, the F1-Score serves as a crucial metric for more accurately assessing the performance of a classification model.

Balanced Accuracy Metric:

Balanced Accuracy calculates the average of the true positive rates for each class. This metric accounts for class imbalance and evaluates the contribution of each class equally. Its mathematical formulation is presented in Eq. 3.

3 $$\:Balanced\:Accuracy=\:\frac{1}{n}\sum\:_{i=1}^{n}\frac{{TP}_{i}}{{TP}_{i}+\:{FN}_{i}}$$

$\:n:$ Number of classes,

$\:{TP}_{i}:$True Positive represents the number of cases in which sample i was correctly classified, 

$\:{FN}_{i}:\:$False Negative represents the number of cases in which sample i was incorrectly classified.

These values are summed across the total number of true positives and false negatives for each class and then divided by the number of classes. As a result, the Balanced Accuracy metric balances the classification performance across classes and evaluates the contribution of each class equally. Thus, it provides a more uniform assessment of the classification model’s performance. Consequently, it is regarded a reliable performance metric for imbalanced datasets.

These metrics are grounded in widely accepted for evaluating classification models and are particularly suitable for assessing model performance in the presence of class imbalance^63^.

Friedman Test:

The Friedman test is a non-parametric statistical method developed by Milton Friedman in 1937^64^. In the context of machine learning and model evaluation, the Friedman test is widely employed to compare the performance of multiple algorithms across different datasets or tasks^65,66^. Unlike parametric tests, the Friedman test does not rely on distributional assumptions, making it particularly suitable for scenarios where the dependent variable is ordinal or where the sample size is small or not normally distributed. The test ranks the models within each dataset or evaluation metric and analyzes these rankings to determine whether statistically significant differences exist among them. Owing to its robustness and simplicity, the Friedman test has become a standard tool in empirical studies involving algorithm benchmarking, particularly in scenarios where evaluation results are obtained under repeated or related experimental conditions.

## Experimental results

In this section, the classification results are presented in tabular form. For each classification model, the performance on both the original dataset and the versions generated through various data augmentation methods is compared. The tables present the results obtained for both training and test data collectively. This study aims to contribute to the process of determining the most effective data augmentation method for a particular machine learning problem.

In the study, the datasets are preconfigured with predetermined training and testing subsets, therefore no further partitioning process was performed. For the vectorization step MiniLM v2 model from Sentence Transformers repository — a Python library for accessing, using, and training state-of-the-art text and image embedding models —was utilized^17^. Data augmentation was performed with an open source package named SMOTE variants^67^, which provides a collection of 85 minority oversampling techniques (SMOTE) for imbalanced learning with multi-class oversampling and model selection features. In this study 31 of these tecniques were employed with their default parameters. The results for each classifier are presented in Tables 4, 5, 6, 7, 8 and 9. Within these tables, bolded values indicate the highest F1-Score and Balanced Accuracy (BA) achieved for each data augmentation method. Classification step was conducted using Python’s Scikit-Learn library with their default parameters^68^, ensuring standardized and reproducible experimental conditions across all techniques.

All experiments were executed on a workstation equipped with an Intel Core i7-12700 CPU, 32 GB RAM, and an NVIDIA RTX 4070 GPU (12 GB). Each experimental run—comprising a specific combination of dataset, classifier, and oversampling method—required approximately 4 to 8 min to complete, encompassing the pipeline of oversampling and classification. Most SMOTE variants executed within acceptable timeframes. However, computationally intensive methods—such as SMOTE-ENN, DE Oversampling, and ADOMS—incurred longer runtimes due to internal operations like density estimation and instance cleaning. Despite these variations, the complete experimental workflow was fully feasible on standard modern desktop hardware, ensuring practical reproducibility and accessibility for future research in this domain.

Table 4 presents the results of the SVM classifier utilized in the study. According to these results, the SVM classifier achieved successful outcomes across nearly all data augmentation methods. Given the already high performance attained with the original data, the contribution of data augmentation methods to SVM performance appears to be somewhat limited. Nevertheless, among the augmentation approaches, Polynom Fit SMOTE and DE Oversampling stand out by delivering superior performance compared to results obtained with the original data.

When the results for the TREC dataset are examined, it can be seen that the training F1-Score on the original dataset is 0.968 with a BA value of 0.959. On the test data, the F1-Score is 0.947 and BA value is 0.936. These results demonstrate the effectiveness of the SVM classifier on the original data. Following the application of various data augmentation methods, a general improvement in performance was observed in the training results. The Minority Driven Oversampling method yielded the highest performance in training (F1-Score 0.996, BA 0.994), but a decrease in performance (F1-Score 0.937, BA 0.919) was observed in the test results, suggesting a possible overfitting. In contrast, the Polynom Fit SMOTE method stood out by providing consistent improvements in both training and test results. It achieved an F1-Score of 0.989 and a BA value of 0.989 in training, while obtaining an F1-Score of 0.950 and a BA value of 0.940 in test data. This method improved overall performance by providing the highest values in test results. Although the Differential Evolution (DE) Oversampling method showed relatively lower performance in training (F1-Score 0.982, BA 0.982), it achieved good performance in test results (F1-Score 0.992, BA 0.982), indicating reduced risk of overfitting and better generalization. Overall, methods like SMOTE and ADASYN produced balanced performance improvement in both training and test results, indicating their robustness across datasets.

Looking at the results for EMOTIONS dataset, it can be seen that the training F1-Score on the original dataset is 0.844 with a BA value of 0.808. On the test data, the F1-Score is 0.658 with a BA value of 0.622. These relatively lower scores suggest that the dataset may be more imbalanced or that class separation is inherently more difficult in this task. Following the application of data augmentation methods, a substantial increase in performance was observed in training performance. The Minority Driven Oversampling method yielded the highest performance in training (F1-Score 0.977, BA 0.971), however, it exhibited notably lower performance on test set (F1-Score 0.643, BA 0.608), indicating a risk of overfitting. Similarly, the Polynom Fit SMOTE method demonstrated high performance in training (F1-Score 0.976, BA 0.976), but its test performance remained low (F1-Score 0.647, BA 0.608).

In contrast, the DE Oversampling method produced the best test set results (F1-Score 0.688, BA 0.678), along with robust training performance (F1-Score 0.961, BA 0.961). This method emerged as the effective data augmentation method with a positive impact on overall performance. Other methods such as SMOTE and ADASYN also significantly improved training performance, but provided limited improvement in test performance compared to the original dataset.

The detailed analysis of Table 4 shows that data augmentation methods exhibit varying effects across different datasets. In the TREC dataset, certain methods (e.g., Polynom Fit SMOTE) achieved a balanced improvement in both training and test performance, whereas others (e.g., Minority Driven Oversampling) only provided substantial improvement in training performance alone, indicating a risk of overfitting.

In contrast, for the EMOTIONS dataset, DE Oversampling achieved the highest test performance, highlighting the dataset-specific effectiveness of augmentation techniques. Overall, while methods that enchanced training performance generally led to improvements in test performance as well, these gains were often limited compared to the performance achieved with the original dataset. This analysis emphasizes the critical importance of selecting appropriate data augmentation strategies based on the specific characteristics and imbalance severity of the dataset. Not all data augmentation methods yield consistent benefits across datasets, and their effectiveness may vary significantly depending on the structure of the dataset. Each row in the result tables corresponds to a distinct SMOTE variant. For each variant, the F1-Score and Balanced Accuracy (BA) are reported separately for the training and test sets.

**Table 4:** Results for SVM classifier.

	TREC				EMOTIONS			
	Train		Test		Train		Test	
	F1-Score	BA	F1-Score	BA	F1-Score	BA	F1-Score	BA
Original	0.968	0.959	0.947	0.936	0.844	0.808	0.658	0.622
SMOTE	0.985	0.985	0.935	0.933	0.968	0.968	0.661	0.633
ADASYN	0.986	0.986	0.936	0.933	0.971	0.971	0.659	0.632
Borderline SMOTE1	0.985	0.985	0.938	0.936	0.959	0.959	0.656	0.626
Borderline SMOTE2	0.985	0.985	0.935	0.933	0.957	0.957	0.666	0.636
SMOTE TomekLinks	0.984	0.984	0.936	0.933	0.968	0.968	0.660	0.634
SMOTE ENN	0.982	0.982	0.937	0.935	0.929	0.922	0.661	0.630
Safe Level SMOTE	0.968	0.968	0.917	0.929	0.926	0.926	0.657	0.644
SMOTE RSB	0.985	0.985	0.935	0.933	0.863	0.831	0.657	0.620
SMOTE IPF	0.985	0.985	0.934	0.931	0.967	0.967	0.661	0.634
Lee	0.984	0.984	0.930	0.927	0.957	0.957	0.647	0.617
SMOBD	0.986	0.986	0.930	0.928	0.964	0.964	0.657	0.627
G SMOTE	0.986	0.986	0.928	0.926	0.968	0.968	0.659	0.632
CCR	0.874	0.873	0.779	0.771	0.538	0.569	0.427	0.429
LVQ SMOTE	0.982	0.982	0.945	0.934	0.944	0.943	0.653	0.619
Assembled SMOTE	0.985	0.985	0.932	0.929	0.968	0.968	0.656	0.631
Polynom Fit SMOTE	0.989	0.989	**0.950**	**0.940**	0.976	**0.976**	0.647	0.608
LLE SMOTE	0.987	0.987	0.926	0.917	0.968	0.968	0.649	0.615
Distance SMOTE	0.987	0.987	0.936	0.934	0.969	0.969	0.661	0.630
SMMO	0.987	0.987	0.939	0.928	0.926	0.907	0.647	0.614
ADOMS	0.982	0.982	0.939	0.937	0.958	0.958	0.682	0.673
MSMOTE	0.980	0.980	0.932	0.930	0.951	0.951	0.657	0.626
DE oversampling	0.982	0.982	0.922	0.928	0.961	0.961	**0.688**	**0.678**
MSYN	0.983	0.983	0.935	0.933	0.961	0.961	0.666	0.645
SVM balance	0.985	0.985	0.933	0.931	0.967	0.967	0.660	0.633
TRIM SMOTE	0.985	0.985	0.933	0.931	0.964	0.964	0.664	0.635
SL Graph SMOTE	0.976	0.976	0.929	0.927	0.953	0.952	0.659	0.630
Random oversampling	0.983	0.983	0.932	0.930	0.965	0.965	0.678	0.673
ROSE	0.984	0.984	0.936	0.934	0.966	0.966	0.681	0.675
Minority driven oversampling	**0.996**	**0.994**	0.937	0.919	**0.977**	0.971	0.643	0.608
Majority data oversampling	0.968	0.959	0.947	0.936	0.844	0.808	0.658	0.622
ClusterOversampling	0.971	0.961	0.943	0.933	0.928	0.907	0.654	0.630

Bold values indicate the best performance.

The results obtained with the k-Nearest Neighbors (KNN) classifier are presented in Table 5. When evaluating the KNN performance on the TREC dataset without any data augmentation, the training F1-Score is found to be 0.774 and the BA value is 0.771. On the test set, the F1-Score is 0.703 and the BA value is 0.726. These results indicate that the KNN classifier demonstrated relatively low performance when trained on the original dataset. Following the application of data augmentation techniques, improvements in classification performance were observed. The Minority Driven Oversampling method achieved the highest performance in training (F1-Score 0.985, BA 0.984), and also yielded the best results on the test set (F1-Score 0.717, BA 0.740). However, the large discrepancy between training and test scores suggests a risk of overfitting. The Polynom Fit SMOTE method produced an F1-Score of 0.769 and a BA value of 0.787 in training, while achieving an F1-Score of 0.661 and a BA value of 0.754 on test data. These results indicatesthat while the method significantly improved the training outcomes, it resulted in only modest gains in test performance. Methods such as SMOTE and ADASYN yielded limited improvements in training but demonstrated relatively stronger improvements in test results. Overall, data augmentation methods enhanced the performance of the KNN classifier. Nonetheless, consistent gap between training and test scores in certain methods highlights the importance of evaluating potential overfitting risks when selecting augmentation strategies.

When the EMOTIONS dataset is examined, it can be seen that the training F1-Score is 0.681 and BA value is 0.637, while the test set yields an F1-Score of 0.577 and a BA of 0.548, when using the original dataset. These relatively low performance values suggest that the dataset is both imbalanced and inherently challenging. Following the application of data augmentation techniques, a substantial performance improvement was observed in training performance. The Minority Driven Oversampling method produced the highest performance in training (F1-Score 0.977, BA 0.970); however, it exhibited lower performance in test results (F1-Score 0.582, BA 0.566), indicating a risk of overfitting.

The MSYN method achieved strong training results (F1-Score 0.845, BA 0.851), and demonstrated improved test performance (F1-Score 0.551, BA 0.626), compared to the original dataset, suggesting better generalization capabilities. Similarly, the DE Oversampling method showed good performance in training (F1-Score 0.908, BA 0.910), but provided limited improvement in test results (F1-Score 0.573, BA 0.595). Overall, data augmentation methods contributed positively to performance on the EMOTIONS dataset; however, some techniques, particularly those yielding disproportionately high training scores, may introduce overfitting risks and require cautious application.

The detailed analysis of Table 5 indicates that the KNN classifier yielded lower performance compared to the SVM classifier, and that data augmentation methods are generally more beneficial for KNN. In the TREC dataset, certain data augmentation methods (e.g., Minority Driven Oversampling) led to substantial improvements in training performance, thereby increasing the risk of overfitting, while offering only limitedited improvement in test performance. In the EMOTIONS dataset, data augmentation methods generally resulted in higher training performance, however enhancements in test performance remained modest. These findings underscore the importance of carefully selecting data augmentation methods for the KNN classifier, as not all techniques exhibit equall effectiveness across different datasets. It becomes evident that identifying the most suitable data augmentation strategy depending on the characteristics and imbalance of the dataset is crucial for optimizing the performance of machine learning models.

**Table 5:** Results for KNN classifier.

	TREC				EMOTIONS			
	Train		Test		Train		Test	
	F1-Score	BA	F1-Score	BA	F1-Score	BA	F1-Score	BA
Original	0.774	0.771	0.703	0.726	0.681	0.637	0.577	0.548
SMOTE	0.790	0.798	0.546	0.670	0.821	0.837	0.474	0.578
ADASYN	0.770	0.780	0.544	0.654	0.820	0.839	0.468	0.574
Borderline SMOTE1	0.788	0.796	0.562	0.668	0.804	0.818	0.494	0.583
Borderline SMOTE2	0.745	0.755	0.543	0.646	0.777	0.794	0.447	0.550
SMOTE TomekLinks	0.755	0.770	0.542	0.663	0.814	0.831	0.466	0.567
SMOTE ENN	0.733	0.751	0.561	0.694	0.722	0.744	0.493	0.577
Safe level SMOTE	0.805	0.811	0.615	0.716	0.825	0.831	0.514	0.587
SMOTE RSB	0.793	0.804	0.568	0.687	0.705	0.660	0.573	0.546
SMOTE IPF	0.792	0.800	0.580	0.687	0.821	0.838	0.471	0.578
Lee	0.805	0.812	0.583	0.703	0.826	0.838	0.516	0.610
SMOBD	0.814	0.820	0.583	0.689	0.862	0.872	0.525	0.616
G SMOTE	0.796	0.803	0.579	0.687	0.816	0.833	0.470	0.576
CCR	0.685	0.698	0.703	0.726	0.455	0.501	0.577	0.548
LVQ SMOTE	0.792	0.800	0.553	0.674	0.836	0.837	0.527	0.562
Assembled SMOTE	0.791	0.799	0.556	0.682	0.817	0.835	0.478	0.583
Polynom fit SMOTE	0.769	0.787	0.661	0.754	0.765	0.782	0.477	0.569
LLE SMOTE	0.753	0.775	0.526	0.655	0.787	0.820	0.457	0.579
Distance SMOTE	0.750	0.764	0.519	0.644	0.766	0.799	0.428	0.560
SMMO	0.661	0.675	0.441	0.562	0.599	0.610	0.342	0.394
ADOMS	0.825	0.831	0.588	0.711	0.896	0.900	0.558	0.605
MSMOTE	0.806	0.811	0.608	0.720	0.816	0.824	0.503	0.578
DE oversampling	0.831	0.836	0.648	0.732	0.908	0.910	0.573	0.595
MSYN	0.824	0.829	0.642	0.738	0.845	0.851	0.551	**0.626**
SVM balance	0.788	0.796	0.586	0.688	0.822	0.839	0.474	0.575
TRIM SMOTE	0.812	0.818	0.596	0.714	0.817	0.832	0.492	0.595
SL Graph SMOTE	0.789	0.795	0.558	0.679	0.800	0.812	0.495	0.590
Random oversampling	0.816	0.820	0.623	0.714	0.854	0.858	0.541	0.591
ROSE	0.815	0.818	0.648	0.735	0.851	0.855	0.536	0.583
Minority driven oversampling	**0.985**	**0.984**	**0.717**	**0.740**	**0.977**	**0.970**	**0.582**	0.566
Majority data oversampling	0.774	0.771	0.703	0.726	0.681	0.637	0.577	0.548
ClusterOversampling	0.766	0.758	0.664	0.666	0.686	0.692	0.448	0.529

Bold values indicate the best performance.

The results of the Decision Trees classifier are presented in Table 6. When evaluating its performance on the TREC dataset, it is observed that the training performance is exceptionally high. Using the original dataset, both the training F1-Score and BA reached a perfect score of 1.000. This trend persisted across all data augmentation methods, with training F1-Score and BA consistently remaining at 1.000. However, a significant drop in performance was observed. The best test results are recorded with ROSE method (F1-Score: 0.514) and the LLE-SMOTE method (BA: 0.551). This pronounced discrepancy between training and test performance indicates that the Decision Trees classifier overfits the training data and fails to generalize to unseen data. These findings suggest that the model memorizes training instances rather than learning generalizable patterns. Moreover, the application of data augmentation methods did not significantly enhance the model’s generalization ability, as evidenced by persistently low test performance across all methods.

The results of the Decision Trees classifier on the EMOTIONS dataset exhibit a similar pattern. While the training F1-Score is 0.997 and BA value is 0.995 on the original dataset, the test results are considerably low, with both F1-Score and BA at 0.284. When data augmentation methods are applied, training performance remains exceptionally high (F1-Score and BA value both 0.999); however, test performance continues to be limited. The highest test results are obtained with the ADOMS method, yielding an F1-Score of 0.304 and a BA value of 0.327. Similar to the observations on the TREC dataset, this outcome indicates that the model is prone to overfitting and fails to generalize the unseen data effectively.

The detailed analysis of Table 6 reveals that the Decision Trees classifier has overfit the training data and failed to represent the test data effectively in both datasets. While the training results are nearly perfect, the test performance remains extremely low, clearly indicating severe overfitting and has poor generalization capability. Although data augmentation methods further enhance the training performance, they are insufficient in improving the test performance. Even the best-performing test results remain at suboptimal levels, suggesting that the Decision Trees classifier is not well-suited for this classification task. These findings highlight the critical importance of carefully assessing both model selection and data augmentation strategies in accordance with the specific characteristics of the dataset and the structure of the problem. In particular, the Decision Trees classifier tends to suffer from overfitting in imbalanced datasets, reinforcing the need to consider more robust alternatives with stronger generalization ability.

**Table 6:** Decision tree classification results.

	TREC				EMOTIONS			
	Train		Test		Train		Test	
	F1-Score	BA	F1-Score	BA	F1-Score	BA	F1-Score	BA
Original	1.000	1.000	0.420	0.432	0.997	0.995	0.284	0.284
SMOTE	1.000	1.000	0.450	0.535	**0.999**	**0.999**	0.299	0.316
ADASYN	1.000	1.000	0.462	0.533	**0.999**	**0.999**	0.278	0.301
Borderline SMOTE1	1.000	1.000	0.457	0.486	**0.999**	**0.999**	0.289	0.301
Borderline SMOTE2	1.000	1.000	0.447	0.511	**0.999**	**0.999**	0.276	0.286
SMOTE TomekLinks	1.000	1.000	0.478	0.537	**0.999**	**0.999**	0.311	0.341
SMOTE ENN	1.000	1.000	0.465	0.539	0.998	0.998	0.289	0.300
Safe level SMOTE	0.990	0.990	0.400	0.452	0.973	0.973	0.289	0.309
SMOTE RSB	1.000	1.000	0.434	0.501	0.997	0.996	0.278	0.278
SMOTE IPF	1.000	1.000	0.456	0.540	**0.999**	**0.999**	0.285	0.307
Lee	1.000	1.000	0.461	0.522	**0.999**	**0.999**	0.289	0.301
SMOBD	1.000	1.000	0.390	0.423	**0.999**	**0.999**	0.296	0.322
G SMOTE	1.000	1.000	0.449	0.505	**0.999**	**0.999**	0.294	0.307
CCR	1.000	1.000	0.400	0.407	**0.999**	**0.999**	0.295	0.296
LVQ SMOTE	1.000	1.000	0.493	0.507	**0.999**	**0.999**	0.292	0.295
Assembled SMOTE	1.000	1.000	0.457	0.515	**0.999**	**0.999**	0.300	0.326
Polynom fit SMOTE	1.000	1.000	0.436	0.456	**0.999**	**0.999**	0.268	0.268
LLE SMOTE	1.000	1.000	0.495	**0.551**	**0.999**	**0.999**	0.269	0.276
Distance SMOTE	1.000	1.000	0.442	0.499	**0.999**	**0.999**	0.294	0.311
SMMO	1.000	1.000	0.456	0.472	**0.998**	0.997	0.269	0.274
ADOMS	1.000	1.000	0.435	0.484	**0.999**	**0.999**	**0.304**	**0.327**
MSMOTE	1.000	1.000	0.457	0.518	**0.999**	**0.999**	0.278	0.293
DE oversampling	1.000	1.000	0.423	0.502	**0.999**	**0.999**	0.293	0.313
MSYN	1.000	1.000	0.463	0.498	**0.999**	**0.999**	0.298	0.308
SVM balance	1.000	1.000	0.449	0.509	**0.999**	**0.999**	0.286	0.306
TRIM SMOTE	1.000	1.000	0.416	0.467	**0.999**	**0.999**	0.298	0.316
SL Graph SMOTE	0.996	0.996	0.477	0.522	0.995	0.995	0.273	0.284
Random oversampling	1.000	1.000	0.465	0.494	0.998	0.998	0.282	0.282
ROSE	1.000	1.000	**0.514**	0.507	0.998	0.998	0.290	0.291
Minority driven oversampling	1.000	1.000	0.421	0.452	**0.999**	0.998	0.290	0.297
Majority data oversampling	1.000	1.000	0.420	0.432	0.997	0.995	0.284	0.284
ClusterOversampling	1.000	1.000	0.453	0.460	0.998	0.997	0.286	0.299

Bold values indicate the best performance.

Table 7 presents the results obtained using the Random Forest classifier. The classifier demonstrated highly effective performance on the TREC dataset. Training results were exceptional across all data augmentation methods, with F1-Score and BA values observed at 1.000. Upon examining test results, it is evident that data augmentation methods led to improved performance compared to the original dataset. The Assembled SMOTE method yielded the highest test scores, achieving an F1-Score of 0.819 and a BA value of 0.839. These findings highlight the robustness of the Random Forest classifier for the TREC dataset, as well as the effectiveness of data augmentation methods in enhancing generalization performance. Furthermore, most other augmentation methods also contributed to notable improvements in test results, underscoring the positive impact of data augmentation strategies when used in conjunction with ensemble-based models such as Random Forest.

The results of the Random Forest classifier on the EMOTIONS dataset reveal a more complex scenario. While the training performance remains notably high, with F1-Score and BA values consistently ranging between 0.999 and 1.000, the test results are considerably lower. The highest test performances were achieved with the SMOTE IPF method, yielding an F1-Score of 0.560, and the SMOBD method, yielding a BA value of 0.554. These relatively low test scores suggest that the model may have overfit the training data and failed to generalize effectively to unseen instances. For the EMOTIONS dataset, most data augmentation methods were insufficient in enhancing test performance and produced suboptimal results.

The detailed analysis of Table 7 indicates that the Random Forest classifier performs effectively on the TREC dataset, but encounters significant overfitting issues on the EMOTIONS dataset. On the TREC dataset, data augmentation methods have led to substantial gains, improving test performance. However, on the EMOTIONS dataset, despite achieving near-perfect training scores, the test performance remained low, indicating poor generalization ability of the model. These findings emphasize that the performance of a given classifier may vary considerably across different datasets, and dataset-specific characteristics play a crucial role in determining classification success. While Random proves to be a robust classifier for the TREC dataset, it demonstrates tendency to overfit when applied to the EMOTIONS dataset. This highlights the importance of careful model selection and hyperparameter optimization tailored to the unique properties of each dataset.

**Table 7:** Classification results for random forest.

	TREC				EMOTIONS			
	Train		Test		Train		Test	
	F1-Score	BA	F1-Score	BA	F1-Score	BA	F1-Score	BA
Original	1.000	1.000	0.733	0.714	0.997	0.996	0.354	0.358
SMOTE	1.000	1.000	0.805	0.817	**0.999**	**0.999**	0.542	0.526
ADASYN	1.000	1.000	0.786	0.806	**0.999**	**0.999**	0.532	0.520
Borderline SMOTE1	1.000	1.000	0.801	0.798	**0.999**	**0.999**	0.501	0.477
Borderline SMOTE2	1.000	1.000	0.791	0.790	**0.999**	**0.999**	0.477	0.457
SMOTE TomekLinks	1.000	1.000	0.809	0.823	**0.999**	**0.999**	0.556	0.539
SMOTE ENN	1.000	1.000	0.788	0.804	0.998	0.998	0.461	0.432
Safe level SMOTE	0.990	0.990	0.780	0.786	0.973	0.973	0.516	0.502
SMOTE RSB	1.000	1.000	0.810	0.818	0.997	0.997	0.370	0.368
SMOTE IPF	1.000	1.000	0.809	0.821	**0.999**	**0.999**	**0.560**	0.543
Lee	1.000	1.000	0.778	0.793	**0.999**	**0.999**	0.526	0.496
SMOBD	1.000	1.000	0.791	0.814	**0.999**	**0.999**	0.544	**0.554**
G SMOTE	1.000	1.000	0.799	0.791	**0.999**	**0.999**	0.522	0.497
CCR	1.000	1.000	0.744	0.727	**0.999**	**0.999**	0.352	0.355
LVQ SMOTE	1.000	1.000	0.784	0.769	**0.999**	**0.999**	0.377	0.369
Assembled SMOTE	1.000	1.000	**0.819**	**0.839**	**0.999**	**0.999**	0.550	0.535
Polynom fit SMOTE	1.000	1.000	0.748	0.730	**0.999**	**0.999**	0.358	0.358
LLE SMOTE	1.000	1.000	0.785	0.763	**0.999**	**0.999**	0.439	0.414
Distance SMOTE	1.000	1.000	0.813	0.811	**0.999**	**0.999**	0.531	0.503
SMMO	1.000	1.000	0.749	0.731	0.998	0.998	0.381	0.382
ADOMS	1.000	1.000	0.797	0.817	**0.999**	**0.999**	0.530	0.515
MSMOTE	1.000	1.000	0.804	0.828	**0.999**	**0.999**	0.530	0.517
DE oversampling	1.000	1.000	0.792	0.800	**0.999**	**0.999**	0.531	0.517
MSYN	1.000	1.000	0.759	0.756	**0.999**	**0.999**	0.495	0.463
SVM balance	1.000	1.000	0.775	0.787	**0.999**	**0.999**	0.559	0.545
TRIM SMOTE	1.000	1.000	0.770	0.766	**0.999**	**0.999**	0.537	0.516
SL graph SMOTE	0.996	0.996	0.782	0.782	0.995	0.995	0.494	0.475
Random oversampling	1.000	1.000	0.804	0.796	0.998	0.998	0.433	0.412
ROSE	1.000	1.000	0.777	0.771	0.998	0.998	0.448	0.422
Minority driven oversampling	1.000	1.000	0.772	0.752	**0.999**	**0.999**	0.433	0.413
Majority data oversampling	1.000	1.000	0.733	0.714	0.997	0.996	0.354	0.358
ClusterOversampling	1.000	1.000	0.734	0.718	0.998	0.998	0.467	0.468

Bold values indicate the best performance.

Table 8 presents the results obtained using the Naive Bayes classifier. For the TREC dataset, without any data augmentation, the training results yielded an F1-Score of 0.755 and a BA of 0.772, while the test results reached an F1-Score of 0.787 and a BA of 0.812. Upon applying data augmentation methods, the highest training performance was achieved using the Minority Driven Oversampling method, with both the F1-Score and BA recorded at 0.832. In the test results, the highest F1-Score was again obtained with the Minority Driven Oversampling method at 0.818, while the highest BA value is observed with the ADOMS method at 0.829. These results indicate that Naive Bayes classifier on the TREC dataset can be effectively enhanced through data augmentation, particularly in improving generalization on the test set.

When the results of the EMOTIONS dataset are examined, training on the original dataset yielded an F1-Score of 0.520 and a BA of 0.564, while test performance resulted in an F1-Score of 0.496 and a BA of 0.542. Following the application of data augmentation methods, the best training outcomes were achieved with the LVQ SMOTE method, which produced an F1-Score of 0.768 and a BA of 0.757. However, in terms of test performance, the highest F1-Score was obtained with the Minority Oversampling method at 0.509, while the highest BA value was achieved with the Random Oversampling method at 0.543. These results indicate that despite notable improvements in training performance, the Naive Bayes classifier demonstrated limited generalization capability on the EMOTIONS dataset. Data augmentation methods did not lead to substantial improvements in test metrics, underscoring a weakness in the model’s ability to generalize beyond the training data.generalization ability.

Overall, the Naive Bayes classifier exhibited strong performance on the TREC dataset. Data augmentation methods — particularly Minority Driven Oversampling and ADOMS — significantly improved test performance. The best test results were obtained with an F1-Score of 0.818 and a BA of 0.829, indicating the Naive Bayes classifier’s strength for the TREC dataset. In contrast, on the EMOTIONS dataset, the classifier demonstrated weaker performance. Despite achieving high training scores, the test performance remained considerably low, and data augmentation methods were largely ineffective in improving generalization. The best test results were obtained with an F1-Score of 0.509 and a BA of 0.543 using Minority Oversampling and Random Oversampling methods. These findings indicate that the effectiveness of the Naive Bayes classifier varies substantially across datasets, and the impact of data augmentation techniques is highly dataset-dependent. While Naive Bayes performed well on the TREC dataset, it showed signs of overfitting on the EMOTIONS dataset. This underscores the necessity of aligning classifier choice and augmentation strategies with the underlying properties of each dataset.

**Table 8:** Naive Bayes classification results.

	TREC				EMOTIONS			
	Train		Test		Train		Test	
	F1-Score	BA	F1-Score	BA	F1-Score	BA	F1-Score	BA
Original	0.755	0.772	0.787	0.812	0.520	0.564	0.496	0.542
SMOTE	0.790	0.788	0.808	0.812	0.656	0.653	0.488	0.489
ADASYN	0.795	0.794	0.806	0.817	0.633	0.631	0.495	0.502
Borderline SMOTE1	0.800	0.799	0.812	0.813	0.692	0.688	0.480	0.474
Borderline SMOTE2	0.787	0.787	0.798	0.810	0.632	0.630	0.483	0.494
SMOTE TomekLinks	0.788	0.786	0.805	0.808	0.657	0.654	0.493	0.492
SMOTE ENN	0.784	0.781	0.812	0.816	0.621	0.625	0.502	0.521
Safe level SMOTE	0.769	0.766	0.783	0.809	0.613	0.610	0.485	0.526
SMOTE RSB	0.789	0.787	0.791	0.788	0.522	0.568	0.489	0.533
SMOTE IPF	0.791	0.789	0.801	0.805	0.655	0.652	0.491	0.490
Lee	0.799	0.798	0.796	0.793	0.707	0.702	0.479	0.467
SMOBD	0.793	0.791	0.801	0.813	0.658	0.655	0.495	0.504
G SMOTE	0.794	0.792	0.806	0.810	0.656	0.653	0.501	0.501
CCR	0.786	0.784	0.651	0.668	0.284	0.414	0.209	0.269
LVQ SMOTE	0.804	0.803	0.775	0.781	**0.768**	**0.757**	0.386	0.367
Assembled SMOTE	0.790	0.789	0.802	0.807	0.645	0.643	0.495	0.497
Polynom fit SMOTE	0.770	0.765	0.622	0.638	0.271	0.394	0.211	0.266
LLE SMOTE	0.795	0.793	0.648	0.664	0.610	0.620	0.294	0.312
Distance SMOTE	0.798	0.796	0.762	0.748	0.677	0.673	0.464	0.439
SMMO	0.801	0.799	0.671	0.682	0.577	0.624	0.400	0.431
ADOMS	0.778	0.777	0.784	**0.829**	0.579	0.578	0.499	0.533
MSMOTE	0.777	0.775	0.797	0.810	0.644	0.641	0.480	0.489
DE oversampling	0.775	0.775	0.771	0.822	0.575	0.575	0.488	0.536
MSYN	0.785	0.783	0.809	0.812	0.676	0.670	0.500	0.509
SVM balance	0.793	0.791	0.808	0.811	0.656	0.653	0.489	0.489
TRIM SMOTE	0.795	0.793	0.797	0.793	0.669	0.666	0.488	0.488
SL graph SMOTE	0.790	0.788	0.799	0.796	0.687	0.683	0.488	0.485
Random oversampling	0.777	0.776	0.778	0.809	0.573	0.572	0.478	**0.543**
ROSE	0.780	0.778	0.782	0.814	0.575	0.574	0.474	0.537
Minority driven oversampling	**0.832**	**0.832**	**0.818**	0.815	0.651	0.701	**0.509**	0.516
Majority data oversampling	0.755	0.772	0.787	0.812	0.520	0.564	0.496	0.542
ClusterOversampling	0.758	0.777	0.777	0.809	0.559	0.599	0.490	0.527

Bold values indicate the best performance.

In Table 9, the results of the Logistic Regression classifier are presented. Overall, the classifier demonstrates strong performance across conditions. Notably the BA values achieved through data augmentation methods are consistently higher compared to the original results.

In the TREC dataset, the original training data yielded an F1-Score of 0.862 and a BA value of 0.842, while the test set achieved an F1-Score of 0.892 and a BA value of 0.872. Among the data augmentation techniques applied, Borderline SMOTE and Minority Driven Oversampling emerged as the most effective methods in improving classification performance.

For EMOTIONS dataset, the LVQ SMOTE method achieved the highest training performance. In the test phase, the Minority Driven Oversampling method yielded the highest F1-Score of 0.601, while the ROSE method produced the highest BA value of 0.644.

The detailed analysis of Table 9 reveals that the Logistic Regression classifier consistently achieved high performance across both datasets. On the TREC dataset, performance was further enhanced through the application of data augmentation techniques. While the test performance on the EMOTIONS dataset also improved with these methods, it did not reach the levels observed on the TREC dataset. These findings suggest that dataset characteristics and the choice of augmentation strategy play a critical role in determining classification effectiveness.

**Table 9:** Logistic regression classification results.

	TREC				EMOTIONS			
	Train		Test		Train		Test	
	F1-Score	BA	F1-Score	BA	F1-Score	BA	F1-Score	BA
Original	0.862	0.842	0.892	0.872	0.626	0.592	0.593	0.566
SMOTE	0.907	0.908	0.888	0.902	0.764	0.765	0.587	0.635
ADASYN	0.910	0.911	0.860	0.889	0.745	0.746	0.572	0.629
Borderline SMOTE1	0.909	0.910	0.897	**0.913**	0.800	0.801	0.581	0.609
Borderline SMOTE2	0.896	0.897	0.878	0.908	0.747	0.749	0.561	0.607
SMOTE TomekLinks	0.905	0.905	0.863	0.894	0.760	0.762	0.581	0.629
SMOTE ENN	0.896	0.897	0.891	0.905	0.721	0.713	0.592	0.612
Safe level SMOTE	0.884	0.884	0.845	0.905	0.730	0.731	0.564	0.612
SMOTE RSB	0.906	0.907	0.887	0.895	0.637	0.604	0.594	0.570
SMOTE IPF	0.907	0.908	0.873	0.892	0.763	0.765	0.584	0.633
Lee	0.908	0.909	0.889	0.897	0.812	0.813	0.594	0.614
SMOBD	0.906	0.907	0.886	0.901	0.761	0.762	0.584	0.635
G SMOTE	0.908	0.909	0.877	0.891	0.765	0.767	0.587	0.637
CCR	0.817	0.818	0.783	0.872	0.414	0.417	0.447	0.497
LVQ SMOTE	0.909	0.910	0.895	0.903	**0.850**	**0.849**	0.598	0.587
Assembled SMOTE	0.909	0.909	0.870	0.896	0.754	0.755	0.584	0.640
Polynom fit SMOTE	0.910	0.911	0.885	0.893	0.845	0.847	0.593	0.621
LLE SMOTE	0.908	0.909	0.868	0.911	0.821	0.824	0.587	0.636
Distance SMOTE	0.909	0.910	0.892	0.899	0.785	0.787	0.593	0.636
SMMO	0.907	0.908	0.846	0.868	0.708	0.693	0.544	0.552
ADOMS	0.900	0.900	0.837	0.885	0.705	0.706	0.571	0.631
MSMOTE	0.895	0.896	0.879	0.894	0.751	0.752	0.574	0.616
DE oversampling	0.901	0.903	0.871	0.896	0.691	0.691	0.567	0.636
MSYN	0.907	0.908	0.888	0.896	0.788	0.789	0.586	0.628
SVM balance	0.908	0.909	0.877	0.898	0.762	0.764	0.590	0.639
TRIM SMOTE	0.908	0.908	0.896	0.896	0.778	0.780	0.588	0.630
SL graph SMOTE	0.899	0.899	0.892	0.909	0.790	0.791	0.587	0.618
Random oversampling	0.906	0.907	0.860	0.890	0.709	0.710	0.574	0.637
ROSE	0.904	0.905	0.854	0.889	0.712	0.713	0.580	**0.644**
Minority driven	**0.949**	**0.939**	**0.899**	0.882	0.826	0.810	**0.601**	0.592
Majority data oversampling	0.862	0.842	0.892	0.872	0.626	0.592	0.593	0.566
ClusterOversampling	0.868	0.847	0.885	0.865	0.657	0.644	0.557	0.581

Bold values indicate the best performance.

When the tables are examined, notable differences are observed in the performance of various classification algorithms across the TREC and EMOTIONS datasets. On the TREC dataset, Decision Trees and Random Forest classifiers generally achieved high success, and their performance was further enhanced by data augmentation methods. These methods had a particularly strong impact on boosting the effectiveness of these models in the TREC context. Naive Bayes and Logistic Regression also yielded competitive results in the TREC dataset, especially when combined with data augmentation techniques, which led to further improvements in classification performance.

For the EMOTIONS data set, the situation is slightly different. In this case, Decision Trees and Random Forests generally exhibited signs of overfitting, leading to lower test results. Similarly, Naive Bayes and Logistic Regression also demonstrated weaker performance in the EMOTIONS dataset. Although data augmentation methods contributed to modest improvements, they were insufficient to fully address the challenges inherent in the EMOTIONS dataset. These findings suggest that the EMOTIONS dataset represents a more complex classification task, highlighting the need to enhance the generalization capacity of the applied models.

### Statistical significance analysis

In comparative machine learning research, it is essential to assess whether performance variations among algorithms across multiple datasets are statistically meaningful or simply attributable to chance. This study utilized the Friedman test to determine whether the observed disparities in classification performance. The level of significance is set at 0.05 for the Friedman test. It means, if the p-Value is less than 0.05, there is a statistically significant difference between the results. Otherwise, there is no significant difference.

Table 10 summarizes the performance comparison of six machine learning classifiers—SVM, KNN, Decision Tree, Random Forest, Naive Bayes, and Logistic Regression—across two benchmark datasets (TREC and EMOTIONS) and two evaluation metrics (F1-Score and Balanced Accuracy (BA)).

**Table 10:** Friedman test results.

			SVM	KNN	Decision tree	Random forest	Naive Bayes	Logistic regression
TREC F1-Score		Average	0.930	0.595	0.448	0.782	0.776	0.875
	Friedman’s test	Mean rank	11.375	3.531	1.563	6.422	6.609	9.500
		Final rank	1	5	6	4	3	2
		p-Value	2.84E-33					
TREC BA		Average	0.926	0.692	0.493	0.782	0.788	0.893
	Friedman’s test	Mean rank	11.375	3.781	1.500	6.156	6.625	9.563
		Final rank	1	5	6	4	3	2
		p-Value	3.83E-33					
EMOTIONS F1-Score		Average	0.653	0.503	0.288	0.477	0.460	0.578
	Friedman’s test	Mean rank	11.313	6.156	1.625	5.719	4.844	9.344
		Final rank	1	3	6	4	5	2
		p-Value	2.46E-29					
EMOTIONS BA		Average	0.627	0.571	0.300	0.463	0.478	0.612
	Friedman’s test	Mean rank	10.688	7.625	1.625	4.484	4.625	9.953
		Final rank	1	3	6	5	4	2
		p-Value	3.25E-31					

In the TREC dataset, both for F1-Score and Balanced Accuracy, Support Vector Machine (SVM) outperforms all other classifiers with the highest average scores (0.930 and 0.926, respectively) and the top mean rank (11.375) in both metrics. This strong performance is reflected in its final rank of 1. Logistic Regression also performs well, consistently securing the second-highest average scores and final ranks. In contrast, Decision Tree yields the weakest performance in both metrics, with the lowest average scores and final ranks of 6. The extremely low p-values (2.84 × 10⁻³³ and 3.83 × 10⁻³³) indicate that the differences in classifier performances are statistically significant, rejecting the null hypothesis that all classifiers perform equally.

For the EMOTIONS dataset, a similar trend is observed where SVM maintains its superior performance, achieving the highest average scores for both F1-Score (0.653) and BA (0.627), as well as the best mean ranks. Logistic Regression again ranks second in both metrics, underlining its robust generalization capabilities across different tasks. Decision Tree remains the weakest classifier with the lowest average scores and worst ranks, suggesting its limitations in handling the complexity or noise present in emotional classification tasks. Interestingly, Naive Bayes and Random Forest show modest but dataset-dependent effectiveness, occupying middle-ground ranks. The p-values (2.46 × 10⁻²⁹ and 3.25 × 10⁻³¹) confirm the statistical significance of the observed performance differences among classifiers on the EMOTIONS dataset as well.

This study highlights how different classification algorithms respond to varying dataset characteristics. Data augmentation techniques have contributed to mitigating class imbalance in the training data and improving overall performance. While several algorithms exhibited strong performance—particularly when combined with data augmentation methods—on the TREC dataset, they encountered greater difficulty when applied to the more complex EMOTIONS dataset. These findings suggest that, depending on the nature of the dataset, the development and application of more sophisticated modeling or augmentation strategies may be necessary to enhance classification performance.

**Table 11:** Classifier-specific summary of oversampling performance.

Classifier	Best performing method	Overfitting risk	General observations
SVM	DE oversampling	Low	Consistent performance on both datasets
kNN	Polynom Fit SMOTE	Moderate	Sensitive to augmentation method
DT	None clearly superior	High	Severe overfitting observed
RF	Assembled SMOTE	Moderate	Strong performance on TREC, overfits EMOTIONS
NB	Minority driven oversampling	Low	Responds well to augmentation
LR	ADOMS	Low	Good balance across datasets

To synthesize the findings across classifiers and datasets, a comparative summary is provided in Table 11. This table highlights the best-performing oversampling method for each classifier, notes observed overfitting tendencies, and summarizes key performance trends identified across both the TREC and EMOTIONS datasets Classifier performance varied substantially across the two datasets. For instance, Random Forest achieved strong results on the TREC dataset but exhibited clear signs of overfitting on EMOTIONS. Naive Bayes and Logistic Regression demonstrated competitive performance when paired with moderately advanced oversampling methods such as ADOMS and LVQ-SMOTE, offering a favorable trade-off between accuracy and computational efficiency. In contrast, Decision Trees displayed pronounced overfitting, even in the presence of data augmentation, suggesting a need for regularization or pruning mechanisms. Support Vector Machines (SVM) consistently achieved high performance, particularly when combined with DE Oversampling or Polynom Fit SMOTE. Although K-Nearest Neighbors (KNN) benefited from data augmentation during training, it produced less stable results on test sets—likely due to its sensitivity to the distribution and positioning of synthetic samples in the feature space. Collectively, these findings underscore the necessity of selecting oversampling strategies not only in accordance with dataset characteristics but also with respect to classifier-specific learning dynamics and generalization behavior.

## Conclusion & future works

This study investigates the impact of data augmentation techniques on text classification tasks. Six different classifiers were applied to the TREC and EMOTIONS datasets to evaluate the effectiveness of various augmentation strategies. The results suggests that the success of data augmentation methods is highly dependent on dataset-specific characteristics, including the degree of class imbalance.

Specific data augmentation methods often yield high F1-Scores and BA values, indicating an overall enchancement in model performance. However, in certain cases, a particular data augmentation technique may outperform others or, conversely, underperform relative to the original dataset. Therefore, selecting the most appropriate data augmentation strategy based on the dataset’s characteristics and problem context is of critical importance. Performance outcomes may vary considerably depending on class distribution and dataset-specific features. As such, conducting empirical evaluations is essential to identify the most effective augmentation approach. Generally, data augmentation techniques are employed to address class imbalance and improve the model’s generalization capability. The findings of this study provide a valuable reference for researchers aiming to select suitable augmentation strategies in the domain of text classification.

Future research stemming from this study may pursue several promising directions. To enhance the robustness and generalizability of text classification models, future efforts should consider expanding dataset diversity by incorporating a broader range of corpora, including domain-specific texts (e.g., legal, academic, and news articles), user-generated content (e.g., social media posts and online reviews), and specialized datasets such as biomedical or multilingual texts. This expansion would allow for a more comprehensive understanding of linguistic variability and context-specific challenges. In addition to traditional classifiers like Support Vector Machines, Naive Bayes, and Random Forest, integrating deep learning architectures—such as Convolutional Neural Networks (CNNs), Recurrent Neural Networks (RNNs), Transformer-based models, and ensemble methods—could yield further insights into the scalability of data augmentation strategies across various learning paradigms. Furthermore, advancing augmentation techniques through the adoption of recent methods like back-translation, contextual word replacement using masked language models, Easy Data Augmentation (EDA), and approaches driven by diffusion models or large language models warrants systematic benchmarking under standardized experimental conditions. Hybrid and multi-stage augmentation pipelines, such as combining synonym replacement with adversarial training or applying GAN-based sequence transformations, also hold promise for improving model performance in the presence of class imbalance and linguistic complexity. Finally, the development of augmentation methods tailored to dataset-specific properties—such as domain sensitivity, class distribution, or data volume—may further enhance model adaptability. For example, entity-aware transformations may be more effective in biomedical contexts than in general-purpose corpora. Collectively, these directions represent fruitful avenues for advancing the field of text classification and data augmentation.

## Data Availability

The dataset used and analyzed in this study is available from the corresponding author upon reasonable request.
